# "The Hospital" Medical Book Supplement.—No. XL

**Published:** 1911-03-18

**Authors:** 


### "THE HOSPITAL"
### MEDICAL BOOK SUPPLEMENT No. XL

CONTENTS.

**Studies in the Psychology of Sex** ... ... ... ... ... ... ... ... 711
**Medicine**—The Principles and Practice of Medical Jurisprudence ... ... ... ... ... 714
Matriculation Directory for January ... ... ... ... ... ... ... 714

### STUDIES IN THE PSYCHOLOGY OF SEX.*

While thanking heartily the American publishers for their comprehensive edition of Dr. Havelock Ellis' great work, of which this is the sixth and concluding volume, the English reader cannot but regret that it has been left to America to father the principal authoritative book on the subject which England has produced. The story of the suppression of the first volume of "Studies in the Psychology of Sex" by the English censor is too well known to need repetition, and the English student can lament only that the English censor has determined that all research on this question must remain the privilege of those acquainted with every other European language but our own. The American publishers deserve thanks at any rate for removing this reproach from our language; our country is still content to bear it without shame, with the indifference, indeed, with that intellectual laziness which is the Englishman's well-known characteristic.

Dr. Havelock Ellis' work is, however, a complete conquest of that, and the prevailing quality of this volume on "Sex in Relation to Society," the final relation in which the sex question has to be considered, is its extraordinary sanity. Any reader who has brought to the delightful task of reading this book a tithe of the culture, the critical faculty of appreciation in literature and art, and of the constructive power of dealing with huge masses of material, which Dr. Ellis acquired in fifteen years of preliminary study, will feel that the opinions it expresses ought to be those of any man of liberal education. There is little startling in the book except its careful digestion of so vast a field of foreign literature. What is startling is the sane criterion which has survived so much study, the author's concentration on the actual state of human need and human weakness, which no ecclesiastical or scientific bias has been allowed to deflect from the straight course of his exposition.

Let us compare this book for a moment with a few of the best known authorities, with Lombroso, with Kraft-Ebing, with Weininger. Lombroso is a specialist, on abnormality, of extensive industry, who has compiled an instructive list of cases, most of them pathological or criminal. As is true of the specialist in general, so of Lombroso. The collector's passion for specimens is balanced by no intensive industry, his deductions are for the most part worthless, he has no philosophy or ideas by which to test his observations. Kraft-Ebing is in a like case; the extensive industry of both is astonishing, but someone was needed to draw a reasonably useful or intelligent moral from their vast array of facts. Young Weininger is a refreshing change from these; he had a dash of insight, there was something of the *à priori* thinker about him, but his work will remain the brilliant essay of a young man, an invaluable protest nevertheless against the tedious inductiveness of his German brothers.

Dr. Ellis falls into neither category. He has shown himself able to walk among the facts as a lion-tamer among lions, without losing his mental balance. He has shown also a still more valuable quality: the realisation that what is wanted on the sex question is not silence, not dogmatism, but a little study and a little common-sense. As Meredith said, the question should be "aired."

A few aphorisms from his chapter on sexual education will make this clear: "All great literature touches nakedly and sanely on central facts of sex"; "the child's desire for knowledge concerning the origin of himself is a perfectly natural, honest, and harmless desire, so long as it is not perverted by being thwarted. A child of four may ask questions on this matter, simply and spontaneously. As soon as the questions are put, certainly as soon as they become insistent, they should be answered, in the same simple and spontaneous spirit, though according to the measure of the child's intelligence and his capacity and desire for knowledge. This period . . . would not in any case be delayed beyond the sixth year (page 48)." Thus the first period of initiation into the origin and reproduction of life is set down for parents, and especially for the mother's guidance. After that the following aphorism practically sums up the case: "Every healthy boy and girl who has reached the age of puberty may be safely allowed to ramble in any good library, however varied its contents." Indeed, we may be excused for adding, the only books which children should be forbidden

* *Sex in Relation to Society*, being Vol. VI. of the above by Havelock Ellis. (Philadelphia: F. A. Davis and Co.)

to read are those which their parents hope most to find under their pillows. Dr. Ellis also recognises that education on sex matters generally is likely to fall, through the laziness or ignorance of parents as a class, more and more into the hands of schoolmasters. For the benefit of those elders, however, who let " I dare not " wait upon " I would " in this matter, the author suggests the assistance of the family doctor, if indeed that invaluable old gentleman is not to be crowded out by the Harley Street surgeon.

As regards books for children this admirable chapter contains further a bibliography that it is hard to find elsewhere, and among those books mentioned with no taint of piety or cant in dealing with the subject are " The Wonder of Life " by Mary Tudor Pole; the works of Mrs. Ennis Richmond; particularly " Almost Fourteen," by M. A. Warren (1892); for teachers: Thomas Beddoes' " Hygeia " (1802, vol. i. essay iv.); Canon Lyttelton's " Training of the Young in Laws of Sex " and his other volumes; also the well-known " Love's Coming of Age," by Edward Carpenter (page 53). This last, however, is surely a little anæmic, and should be followed by the young reader with " Joseph Andrews " or " Tom Jones." This chapter concludes by remarking " What has been said about literature applies equally to art," and its successor on " Sexual Education and Nakedness " may be summed up in a remark of Höller, which is quoted approvingly, that " he who has once learnt to enjoy peacefully nakedness in art will be able to look on nakedness in Nature as on a work of art." How few modern people are able peacefully to enjoy either art or nature we are reminded by the recent public outcry against Mr. Eppstein's Strand statues on the British Medical Association's building. On the obvious fact that scant clothing is provocative while nudity is unexciting sexually, Dr. Ellis truly says: " If the conquest of sexual desire were the first and last consideration of life, it would be more reasonable to prohibit clothing than to prohibit nakedness."

" The Valuation of Sexual Love " forms the subject of another chapter in which the ascetic view of sex and the constituents of Love receive an interesting analysis. The ascetic view was summarised by St. Augustine in a well-known phrase, and, while admitting the full force of the arguments which Dr. Havelock Ellis marshals against it, we must admit that, whether due or not to the survival of the Church's contempt for the flesh, these arguments remain in some odd way unconvincing. Not Havelock Ellis, not Walt Whitman himself, can conquer by mere intellectual argument the innate squeamishness with which the dual functions of the sex organs have been regarded always, in however slight a degree, by any human being who has ever thought much about them. One can rejoice in health without necessarily rejoicing in all its processes. Metabolism satisfies the scientific curiosity of the mind, it does not satisfy the æsthetic desires of the soul. We must accept the facts of Nature cheerfully, without brooding upon them. Nothing is gained by a squeamish protest, but squeamishness is nevertheless a part of human nature too; and the wise man will take courage from the fact that life itself, like the metabolism of nutrition and decay, will not bear much thinking about. We must know and accept, we are not obliged to rejoice in every detail of Nature's processes: these are to be obeyed cheerfully, but are not meant to be delighted in for their own sake. Metabolism for metabolism's sake is a mad doctrine, of no more, though of no less, value than the same protest against prudery in another sphere, the cry of art for art's sake at the end of the nineteenth century.

The old question, What is love? has provoked Dr. Ellis to say some interesting things, and from them we take the following analysis: " Love, in the sexual sense, is, summarily considered, a synthesis of lust (in the primitive and uncoloured sense of sexual emotion) and friendship. It is incorrect to apply it to any variety or combination of varieties of friendship. There can be no sexual love without lust; but, on the other hand, until the currents of lust in the organism have been so irradiated as to affect other parts of the psychic organism—at least the affections and the social feelings—it is not yet sexual love. Lust, the specific sexual impulse, is indeed the primary and essential element in this synthesis, for it alone is adequate to the end of reproduction, not only in animals but in men" (page 133). Love, he continues, is the flower which springs from the root of sexual desire: in both, sex is phanergamous, in both the root lies hidden in the primary desire or earth from which they spring. On chastity the same breadth of view is to be found. He says: " If chastity is merely a fatiguing effort to emulate in the sexual sphere the exploits of professional fasting men, an energy using up all the energies of the organism and resulting in no achievement greater than the abstinence it involves, then it is surely an unworthy ideal. If it is a feeble submission to an extreme conventional law which there is no courage to break, then it is not an ideal at all. If it is a rule of morality imposed by one sex on the opposite sex, then it is an injustice and provocative of revolt." Of the chastity still demanded as the stock feminine virtue he remarks, in the words of Westermarck, that " marriage by purchase has raised the standard of female virtue " on the significant ground that the seduction of an unmarried girl " is chiefly, if not exclusively, regarded as an offence against the family of the girl," there being " no indication that it is even held by savages that any wrong has been done to the girl herself." Some fascinating pages then follow on the literature of chaste love, from the " Discourse to those who keep virgins in their houses " of St. Chrysostom to the story of " The two lovers in Auvergne " by Gregory of Tours. The romantic view of chaste love found here and in the Acta Sanctorum, of which this romance may be regarded perhaps as the last, then developed into the more modern view of the chastity of marriage love, expounded first in " Aucassin and Nicolette," and developed to its latest and extremest verge in some of the erotic and mystical poems of Coventry Patmore. The connection between the physical virtue of virginity and its spiritual counterpart in chastity is then described, the former delaying the latter's recognition as being not abstinence, but a reasonable exercise of normal functions. Dr. Ellis adds, by the way, that " chastity has a more special value for those who cultivate the arts " (page 172). " We may not always be inclined to believe the writers who have declared that their verse alone is wanton, but their lives chaste. It is certainly true, however, that a relationship of this kind tends to occur. The stuff of the sexual life, as Nietzsche says, is the stuff of art; if it is expended in one channel it is lost for the other; " again " there is an antagonism between extreme brain vigour and extreme sexual vigour " he says.

Dr. Ellis then shows the chaotic state of written opinion on what is meant by and what is the value of sexual abstinence, and, with Rohleder adopts as his conclusion the practice of a world that has been unwillingly led from the first by ecclesiastical or other special pleaders, namely, that " permanent abstinence is unnatural, and has never existed," the balance between it and license being held by " the biological fact that the act of sexual union is the satisfaction of the erotic needs, not of one person, but of two."

We are forced to omit consecutive reference to the history of prostitution from its religious origin in the orgy to the wrong attempts to-day to regulate it by registration, wrong because experience has shown them to be unavailing in their aim, the reduction of the spread of venereal diseases. The curtailment of these must be sought for elsewhere: first, by a search into the causes of prostitution, which Dr. Havelock Ellis finds, as one expects, to reside in the economic effects of poverty, and more especially in the legal monogamy of the present system of marriage. Schopenhauer remarked that " prostitutes are human sacrifices on the altar of monogamy." Bernard de Mandeville, that forgotten writer, whose " Fable of the Bees " certainly deserves to be added to Messrs. Dent's Everyman's Library at the earliest date, is also quoted to show how many people of importance, including St. Augustine and Aquinas, have held that prostitutes are like drains in this, that, unpleasant in themselves, they insure the purity of our houses. While admitting, however, that prostitution has been condoned as a price worth paying for the ecclesiastical form of monogamic marriage, the problem of venereal disease has forced itself gradually upon the reluctant community. The best methods of reducing the virulence of this group of maladies are summarised by Dr. Ellis under four heads, the first of which is discussed on the principle that " the first step in dealing with a contagious disease is to apply to it the recognised principles of notification," as illustrated practically in the system which obtains in Scandinavia. There, " the sanitary authorities are now exclusively medical," and, which is even more important, " everyone, whatever his social or financial position, is entitled to the free treatment of venereal disease." Free dispensaries are established, which are open in the evenings for advice, and, as a necessary concomitant of the free facilities given to the diseased subject, every man or woman is held responsible for the diseases he or she communicates; and the transmission of syphilis is made the ground for an action for damages, and for divorce in the case of married persons; the transmitter is liable to imprisonment. Even in England, we learn on page 349, " it is now well settled that the infection of a wife by a husband may be held to constitute the legal cruelty, which must be proved, in addition to adultery, before a wife can obtain a divorce from her husband." Finally, education in sexual hygiene must follow to make the above regulations fully enforceable, and to protect the ignorant, so far as that is possible. In Germany free lectures on venereal disease are given, and " should be free to all who have attained the age of sixteen."

Perhaps sufficient quotation has been given to enable the reader to judge of the scope which Dr. Havelock Ellis has included under the title of the present volume. It seemed fairer to the author to allow him to state as far as possible in his own words the conclusions he had reached on at least many divisions of his subject, than to attempt a rapid summary of this great work as a whole. No brief review can do it justice, and, as will be seen, the conclusions themselves lose half their value when unaccompanied by the reasoning which has led to their adoption.

It is the point of view of the author, that sanity and good sense which we noticed at the outset, that is the most valuable and the most prominent quality of the work as a whole. The inculcation of that on the lay, as much as on the medical, public, should be its effect on all intelligent readers: it is more important that they absorb something of Dr. Ellis' sanity on the sex question, than that they should echo all his conclusions. The function of the medical profession is clear, though it is one that has been shamelessly neglected: it is to be the pioneer of honesty in sex matters, to bring a little light and air into the stultifying nonsense-crammed atmosphere of the respectable home. Vulgarly speaking, knowledge of the mysteries of sex and procreation is supposed by the ordinary man to be the unique specialty of the doctor. The doctor then is an enemy of the people if he does not explode this vulgar opinion in the interests of common-sense and hygiene. Alas! it is the doctors themselves who want education so often; it is we, who, having received a scientific education, continue to remain insensible to the scientific spirit, narrow dogmatists, jealous of the secrets of our trade. Doctor and layman alike cannot fail to benefit from reading this honest book, which is direct and frank from first to last, illuminated also by a lucid style and the charm that always resides in the writings of a man who has moved over other fields besides his own, bringing art, literature, and history under contribution to his subject. All three, and a Shakespearean sympathy with human beings as such, were necessary for the adequate treatment of so vast a subject as Sex in Relation to Society. They have made it fascinating, and the work which embodies them is certain to be regarded as the standard volume in English, the classic on a subject that it has been the province of foreigners hitherto to explore.

### MEDICINE.

The Principles and Practice of Medical Jurisprudence. By the late Alfred Swaine Taylor, M.D., F.R.S. Sixth edition. Edited, revised, and brought up to date by Fred J. Smith, M.D. 1910. (London : J. and A. Churchill, 7 Great Marlborough Street. Two vols., £2 2s. net.)

Incontestably the premier work in the English language on the subject with which it deals, Taylor and Stevenson, or as it must now be called Taylor and Smith, challenges comparison with the best foreign works of the same class, and with this, the sixth edition, in front of us, we are compelled to turn to what is, when all is said and done, the final work on the subject of medical jurisprudence, the new edition of Casper's monumental monograph. And let us say at once that the English work stands this comparison very admirably. We miss, it is true, the critical acumen and the elaboration of the jurisprudential part which one may reasonably expect in such a work as this and which are marked features of the German work. The reason for the latter omission, if we may say so, we think lies in the fact that Dr. Smith is not a lawyer; he does not possess the legal mind that judges, or ought to judge, dispassionately; he allows himself to be far too much interested in his subject to write objectively. This is well seen in the section dealing with anæsthetics, where he allows himself to criticise adversely (and in our opinion with irrelevant attempts to raise a smile from his readers) the list of questions drawn up by the coroners for answer in cases of a fatality under anæsthetics. It is true he quotes from a lecture delivered to students, in which such irrelevancies are permissible, but we have to deal with the section as part of a standard book, and it appears to us be out of place. Again, in the sections dealing with sexual crimes he goes little beyond the narrow limits which the editor of the previous issues allowed himself. In a work dealing with such an important science as medical jurisprudence there is no justification for squeamishness and prudery; we have to admit, once and for all, however distasteful the admission may be, that we can no longer regard this special matter to which we have alluded as one "of which far too much has been made by Continental authorities." We may differ as to the extent to which the law should take cognisance of the more recent work on this "unsavoury subject," but as medical men—and this book is obviously intended for medical men—it is imperative that the question should be fully and adequately discussed. With this little tilt against Dr. Smith's editing, we have finished so far as carping criticism is concerned. Coming now to the work itself, we can congratulate Dr. Smith on the very able manner in which he has dealt with the new subject-matter which the wealth of recent citable cases has brought to his notice. Dr. Stevenson brought the 1891 edition thoroughly up to date; the present editor five years ago improved the text-book by adding a series of new cases and by revising it. In this edition there is a further improvement, due mainly to the additions of new, and the omissions of antiquated, matter. We notice with gratification the exclusion of certain illustrations; in such a work woodcuts are of comparatively little value, unless they are exceptionably able colour-reproductions, and we should like to see the number of blocks still further reduced. It is true some authorities—notably Hoffman—lay great stress on the importance of sketches, but we fail to see the advantage of an illustration such as, for example, appears on p. 735 (suicidal hanging). That particular block has perhaps some historical association, but as a typical specimen illustration of the widely diversified manner in which a suicide may commit strangulation it is worthless. On the other hand, we are glad to find that the editor has increased the number of cases in brevier type. They comprise an important feature of the book, and are exceedingly valuable for reference purposes. The text of the two volumes is wholly admirable, and in particular the sections dealing with trade-diseases, lunacy (which has been excellently condensed), wounds, and presumption of death are very clear and full in the first volume; while the second volume, —with the reservations already mentioned,—is equally good. A great deal of new matter has been incorporated owing to the considerable development in medico-sociological work, and the more recent Acts which are of special interest to the practitioner are fully discussed. One of the most important additions to the new edition is the exhaustive discussion on the relative question of compensation claims, the literature of which has already assumed prodigious dimensions. Here it is summarised and discussed in its due relation to legal medicine, and this section of the book is particularly praiseworthy on account of the able manner in which the editor has grappled with the whole intricate subject.

No medical man should be without a book on medical jurisprudence. At any moment he may be called upon to face a situation in which it is imperative that he should be in a position to refer to some authoritative opinion, or to assist his memory by a special case or dictum. In his student days the subject is usually indifferently weighed, on the supposition that only the expert will have to deal with difficulties, and that the ordinary practitioner is exempt from examination as an expert witness. There can be no greater mistake than this. Circumstances may turn the humdrum of daily practice into a period of stupendous excitement, during which the medical man may be called upon to give an expert opinion on a medico-legal case. If the practitioner is not prepared to meet such an emergency he fails in his obligations to the community and to himself. It is therefore necessary that he should have at hand some really reliable manual on medical jurisprudence to which he can refer when occasion demands. As we have said, Taylor and Smith is *facile princeps* among English text-books on the subject, and this new edition is a distinct advance upon its predecessors. The practitioner can therefore do no better than invest in it. The two guineas that the work costs will be money well spent, for it will purchase a couple of volumes that are not only hugely interesting reading in themselves but which are undoubtedly the most up-to-date and reliable exposition of the manifold aspects of legal medicine that exists in the English language.

---

Matriculation Directory for January. "University Tutorial Series." (Burlington House, Cambridge.)

Like everything that emanates from this well-known coaching college, this is an excellent *vade mecum* for the student who is attempting the London Matric. The papers for January are fully worked out, and explanatory notes accompany them. From the little book we gather that the last set of examination papers set were not more than usually "stiff"; from a glance at the papers given we are inclined to believe that many of the questions were more than ordinarily stupid. The "Directory" is bound to be exceedingly useful to any student who is preparing for the Matric.

